# The association between social media use and mental health among adolescents and young adults

**DOI:** 10.1192/j.eurpsy.2022.347

**Published:** 2022-09-01

**Authors:** B. Koronczai, Z. Demetrovics

**Affiliations:** 1 Institute of Psychology, ELTE Eötvös Loránd University, Developmental And Clinical Child Psychology, Budapest, Hungary; 2 University of Gibraltar, Centre Of Excellence In Responsible Gaming, Gibraltar, Gibraltar

**Keywords:** Adolescents, problematic social media use, young adults, mental health

## Abstract

**Introduction:**

The associations of problematic social media use, the special use of image-based social media (photo editing, following celebrities) and mental health (body dissatisfaction, self-esteem, depression) have been established (e.g. Yurdagül et al., 2019; Gioia, Griffiths, Boursier, 2020; Lowe-Calverley and Grieve, 2021). The links may be explained with the theory of social comparison and self-objectification.

**Objectives:**

Testing theory-oriented hypotheses related to image-based social media use and body dissatisfaction, gender specifically, among adolescents and young adults.

**Methods:**

Three surveys have been conducted with convenience sampling: (1) 117 Hungarian university students in person (mean age=22.4, SD=2.9, 79% female), (2) 383 high school students in person (mean age=16.5, SD=1.2, 58% female); (3) 124 Israeli adolescents online (mean age=16.8, SD=2.7, 68% female).

**Results:**

(1): The tendency of modifying body image in social media (the frequency of modifying pictures, the use of filters) mediates the association between body shame and problematic social media use. Physical appearance social comparison mediates the association between self-related negative emotions and attitude (low self-esteem+ineffectiveness) and problematic social media use. (2): The technology-based social comparison mediate the association between muscle checking and problematic Instagram use among boys. (3) Physical appearance social comparison mediates the association between the frequency of following celebrities and body dissatisfaction among girls, but not among boys.

**Conclusions:**

During the use of image based social media, social comparison and the exposure to the beauty standards may lead to poorer mental health, which could result in problematic social media use as maladaptive coping.

**Disclosure:**

No significant relationships.

